# Nanostructuring with Structural-Compositional Dual Heterogeneities Enhances Strength-Ductility Synergy in Eutectic High Entropy Alloy

**DOI:** 10.1038/s41598-019-47983-y

**Published:** 2019-08-08

**Authors:** S. R. Reddy, S. Yoshida, T. Bhattacharjee, N. Sake, A. Lozinko, S. Guo, P. P. Bhattacharjee, N. Tsuji

**Affiliations:** 1 Department of Materials Science and Metallurgical Engineering, IIT Hyderabad, Hyderabad, India; 2 Department of Materials Science and Engineering, Kyoto University, Kyoto, Japan; 3 Elements Strategy Initiative for Structural Materials (ESISM), Kyoto University, Kyoto, Japan; 4 Industrial and Materials Science, Chalmers University of Technology, SE-41296 Gothenburg, Sweden

**Keywords:** Materials science, Metals and alloys

## Abstract

A lamellar (L1_2_ + B2) AlCoCrFeNi_2.1_ eutectic high entropy alloy (EHEA) was severely deformed by a novel hybrid-rolling process. During hybrid-rolling, the deformation was carried out in two stages, namely cryo-rolling followed by warm-rolling at 600 °C. The strain (ε) imparted in each of these steps was identical ~1.2, resulting in a total strain of ε~2.4 (corresponding to 90% reduction in thickness). The novel processing strategy resulted in an extremely heterogeneous microstructure consisting of retained lamellar and transformed nanocrystalline regions. Each of these regions consisted of different phases having different crystal structures and chemical compositions. The novel structure-composition dual heterogeneous microstructure originated from the stored energy of the cryo-rolling which accelerated transformations during subsequent low temperature warm-rolling. The dual heterogeneous microstructure yielded an unprecedented combination of strength (~2000 MPa) and ductility (~8%). The present study for the first time demonstrated that dual structure-composition heterogeneities can be a novel microstructural design strategy for achieving outstanding strength-ductility combination in multiphase high entropy alloys.

## Introduction

Ultrahigh strength materials with adequate ductility is a critical requirement for advanced structural applications. However, concurrent improvement of strength and ductility remains a formidable challenge^1^. A novel approach for managing strength-ductility simultaneously has evolved recently by designing materials with heterogeneous microstructures consisting of different constituents or domains with different hardness/strength^2,3^. A wide variety of heterogeneous microstructures including bimodal grained structure^4–6^, harmonic structure^7^, gradient nano-grained structure^8–12^, nano-domain structure^13^ and nano-twinned grains^14^, laminate structure^15,16^, heterogeneous lamellar microstructure^17,18^ and dynamically reinforced heterogeneous structure^19^ have been reported recently.

Multiphase materials are evidently suited for tuning their mechanical properties by means of heterostructuring. Recently, the emergence of multicomponent high entropy alloys (HEAs) has opened up the massive composition space for developing alloys with novel microstructures and properties^20–26^. Eutectic HEAs (EHEAs), first proposed by Lu *et al*.^27^, are a special class of HEAs having promising mechanical properties in the as-cast condition. In particular, the AlCoCrFeNi_2.1_ nano-lamellar EHEA consisting of soft L1_2_/FCC and hard B2 phases has attracted considerable attention due to their attractive mechanical properties over a wide temperature range^28,29^ and ample opportunities for further tailoring their microstructure and properties by thermo-mechanical processing (TMP)^30,31^. Bhattacharjee *et al*.^32^ showed that heavily cryo-rolled and annealed AlCoCrFeNi_2.1_ EHEA develops a complex heterogeneous microstructure consisting of retained lamellar and coarse non-lamellar regions. The individual domains again consisted of multiscale architecture ranging from ultrafine recrystallized FCC grains to coarse recovered B2 grains, which resulted in simultaneous enhancement in strength and ductility. Shi *et al*. have shown that the properties of the EHEA can be further improved by tuning the inherited lamellar microstructure of the EHEA^33^.

It is important to recognize that the phase compositions in these HEAs can be metastable, so that novel processing routes can accentuate precipitations or transformation of completely new phases even in single phase HEAs^34^. Therefore, significant potential for the control of compositional heterogeneities exists in addition to microstructural heterogeneities. Although this novel approach seems rather exciting, surprisingly it has not been demonstrated as yet.

Here, we have investigated the effect of a novel TMP route combining cryo-rolling followed by warm-rolling (henceforth referred to as hybrid-rolling) on the AlCoCrFeNi_2.1_ EHEA. The motivation is to exploit the stored energy of the cryo-rolling step to accelerate the transformation during warm-rolling at relatively low deformation temperatures, eliminating structural coarsening. The novel processing strategy results in a novel heterogeneous nanostructure combining unprecedented strength and ductility. We further envisage that this novel processing strategy will open up unexplored avenues for developing a new class of HEAs with advanced properties.

## Results

**Figure 1:**
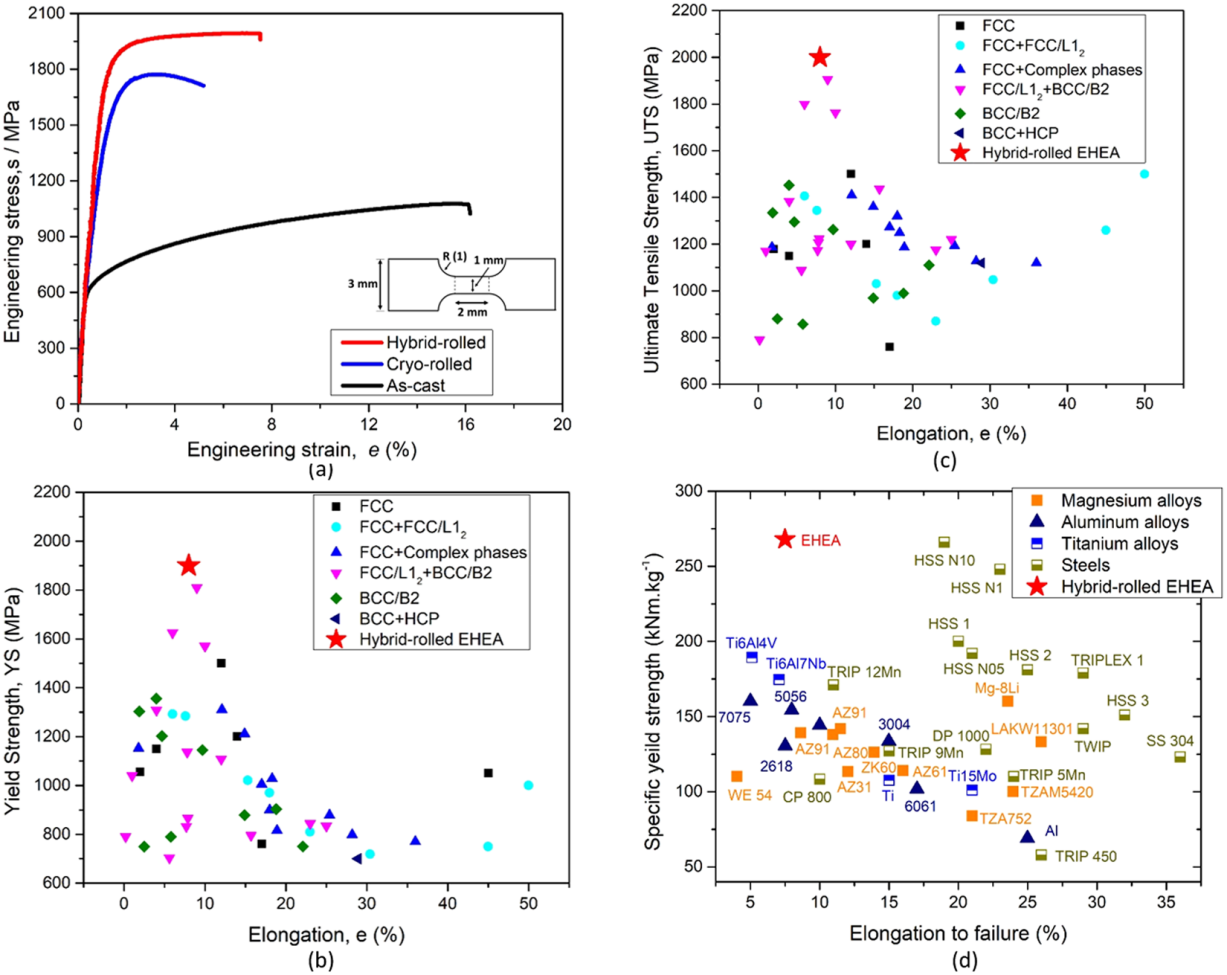
(**a**) Engineering stress-strain curves of the EHEA in the as-cast, cryo- and hybrid-rolled conditions. The dimensions (in mm) of the tensile specimens are shown inset. The (**b**) ${\sigma }_{YS}$ vs e_f_ and (**c**) ${\sigma }_{UTS}$ vs e_f_ plots compare the mechanical properties of the hybrid-rolled EHEA with selected HEAs (the relevant references are included in the Table 1 of the supplementary data). (**d**) Compares the specific strength of the hybrid-rolled EHEA with selected commercial^45^ and advanced alloys (the relevant references are included in the Table 2 of the Supplementary Data).

Figure 1(a) illustrates the engineering stress vs strain plots of the EHEA in the as-cast, only cryo-rolled and hybrid-rolled to 90% reduction in thickness (ε ~ 2.4). The as-cast EHEA shows low yield strength (*σ*_*YS*_) ~600 MPa but possesses high ultimate tensile strength (${\sigma }_{UTS}$) ~1100 MPa and appreciable elongation to failure (*e*_*f*_ ~ 17%). Cryo-rolling results in remarkable increase in strength (*σ*_*YS*_ ~ 1600 MPa and ${\sigma }_{UTS}$ ~ 1750 MPa), although the ductility, particularly uniform elongation (~3%) is diminished. Remarkably, The hybrid-rolled EHEA enjoys outstanding mechanical strength (*σ*_*YS*_ ~ 1900 MPa and ${\sigma }_{UTS}$ ~ 2000 MPa), yet maintains appreciable uniform elongation (*e*_*f*_ ~ 8%); more than twice of the cryo-rolled material. Evidently, the hybrid-rolled EHEA shows simultaneous increase in strength and ductility when compared to the cryo-rolled EHEA. When compared to the as-cast EHEA, the *σ*_*YS*_ increases more than three times and the ${\sigma }_{UTS}$ is nearly doubled.

The outstanding mechanical properties of the hybrid processed EHEA are compared with other HEAs in the $${\sigma }_{YS}\,vs\,{e}_{f}$$ (Fig. 1(b)) and $${\sigma }_{UTS}\,vs\,{e}_{f}$$ (Fig. 1(c)) plots. Figure 1(b,c) include HEAs with strength greater than 700 MPa. Clearly, the EHEA possesses the highest YS and UTS amongst the HEAs reported so far. The specific strength ($${\sigma }_{YS}/\rho $$, where ρ is the density) vs *e*_*f*_ (Fig. 1(c)) compares the EHEA (ρ ~ 7.1 *kg*.*m*^−3^) with several commercial and advanced alloys. The hybrid-rolled EHEA not only shows the highest specific strength (~270 $$kNm.k{g}^{-1})$$, but also is at least 50% more than other commercial alloys (e.g. Ti-6Al-4V or Ti-6Al-7Nb) having similar elongation. When compared to high pressure torsion (HPT) processed alloys^35^, the novel hybrid processing route enjoys the distinct advantage of bulk processing, thus overcoming the limitations of small specimen size that can be fabricated by HPT.

**Figure 2:**
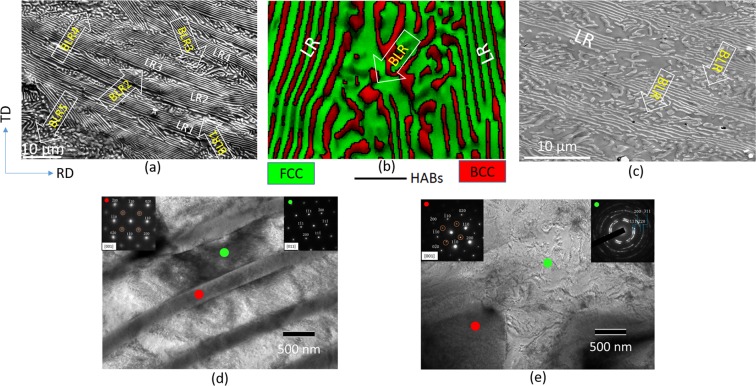
(**a**) SEM micrograph and (**b**) EBSD map of the as-cast EHEA showing the LRs and BLRs. (**c**) shows the SEM micrograph after the Stage I deformation by cryo-rolling (ε = 1.2). (**d**) and (**e**) show the TEM micrographs of the LRs and BLRs, respectively (the SADPs are shown inset), after the Stage I deformation by cryo-rolling (ε = 1.2).

The as-cast EHEA (Fig. 2(a)) shows perfect lamellar regions (LRs) consisting of L1_2_ and B2 lamellae. Imperfect areas in the form of broken lamellar regions (BLRs) are sandwiched between the LRs. The volume fractions of the FCC and B2 phases are ~65% and 35%, respectively, while the average lamellae thickness of the two phases are ~640 ± 40 nm and 240 ± 15 nm, respectively. The thickness and morphology of the BLRs differ considerably. The BLR1 and BLR2 show slightly elongated morphology and thickness ~2–4 µm, whereas BLR4 and BLR5 depict more globular morphology and higher thickness ~8–10 µm. The EBSD map of the BLR (Fig. 2(b)) shows that the two phases (FCC: green, BCC: red) are separated by high angle boundaries (HABs) (black lines). The HABs are absent within the individual constituents. The LRs and BLRs are retained after Stage I deformation by cryo-rolling (Fig. 2(c)). The TEM micrograph obtained from a typical LR is shown in Fig. 2(d). The SADP (upper-right inset) obtained from the green circled spot in Fig. 2(d) shows the zone axis (ZA)//[011] pattern without superlattice reflections, thus confirming the disordered FCC structure. The SADP (upper-left inset) obtained from the red circled spot in Fig. 2(d) shows the ZA//[001] BCC pattern containing superlattice reflections (indicated by circles), indicating ordered B2 phase. The TEM micrograph obtained from the BLR is shown in Fig. 2(e). The upper-right inset (obtained from the green circled region in Fig. 2(e)) shows FCC ring pattern indicating deformation induced disordering and nano-crystallization of the L1_2_. In contrast, the SADP shown in the upper-left inset in Fig. 2(e)) (obtained from the red circled spot in Fig. 2(e)) shows the ZA//[001] BCC pattern featuring superlattice reflections, indicating ordered B2 structure.

**Figure 3:**
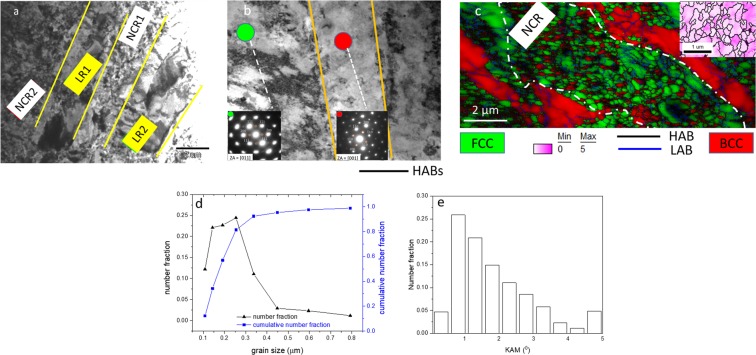
(**a**) Large area TEM micrograph of the hybrid- rolled EHEA showing LRs and adjacent NCRs; (**b**) shows a magnified view of the LR. The SADPs of the L1_2_ (green circle) the and B2 (red circle) present in the LRs are shown inset; (**c**) shows the EBSD map of the FCC (green) and BCC (red) in the NCR. The inset in (**c**) shows KAM map of a selected region in the NCR. (**d**) and (**e**) show the grain size and KAM plots, respectively in the NCR.

The TEM micrograph of the hybrid-rolled EHEA shows remarkable heterogeneities featured by LRs and adjacent nearly equiaxed nanocrystalline regions (NCRs) (Fig. 3(a)). The SADP (inset in Fig. 3(b)) obtained from the green circled spot in Fig. 3(a) shows the ZA//[011] FCC pattern with distinct superlattice reflections (highlighted by the enclosed circles), confirming the ordered L1_2_. The SADP shown inset in Fig. 3(b) (obtained from the red circled spot in Fig. 3(a)) shows the ZA//[001] BCC pattern containing superlattice spots (highlighted by the enclosed circles), thus clearly revealing the ordered B2. Therefore, the ordered phases in the as-cast EHEA are retained in the LRs.

The NCRs sandwiched between the LRs show varying thickness. The region marked as NCR1 is only ~2 µm thick, while the region marked NCR-2 is several microns wide. Figure 3(c) shows a NCR (enclosed by dotted loop). The NCR shows a mixture of equiaxed nanocrystalline FCC (green) and BCC (red) grains. The grains are separated by HABs, although some of these grains show internal low angle boundaries (LABs) (blue lines). Figure 3(d) shows a large fraction of grains is in the nanocrystalline range (average size ~240 nm). The kernel average misorientation (KAM) plot (Fig. 3(e)) shows that ~70% of the grains are in a deformed state (KAM ≥ 1°), also corroborated by the LABs network (Fig. 3(c)).

**Figure 4:**
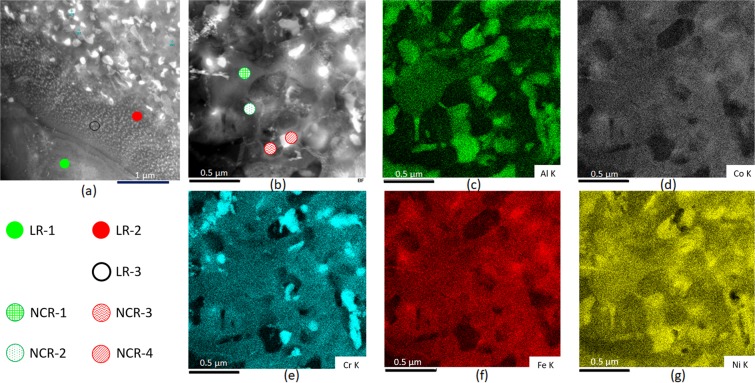
(**a**) HAADF-STEM image showing LR and adjacent NCR.; (**b**) shows a magnified view of the NCR. The composition mapping of the area shown in (**b**) reveals the elemental distribution of (**c**) Al, (**d**) Co, (**c**) Cr, (**f**) Fe and (**g**) Ni in the NCR. The chemical composition of the individual phases are summarized in Table 1.

Figure 4(a) shows a HAADF (high angular annular dark field imaging) –STEM (scanning transmission electron microscopy) image of a composite region containing LRs and adjacent NCR. The phases present in these two regions are prefixed by LRs and NCRs, respectively. The crystal structure and chemical compositions of phases present are summarized in Table 1. The constituents of the LRs, namely LR-1 and LR-2 have the L1_2_ (green circle) and B2 (red circle) structures, respectively, as confirmed by the SADP analysis (Fig. 3(b)). The B2 lamella also contains profuse nano-precipitates (LR-3; open circle in Fig. 4(a)) retained from the as-cast EHEA^30,31^. The chemical compositions of the L1_2_ and B2 phases present in the LRs of the hybrid-rolled EHEA appear very similar to their respective compositions in the as-cast EHEA (shown in parenthesis below the respective phases in Table 1).

**Table 1:** Composition of the phases in the hybrid-rolled EHEA. The figures in the parenthesis show the composition of the corresponding phases in the as-cast EHEA.

Phases	Elements (at.%)					
Designation	Structure	Al	Co	Cr	Fe	Ni
**LR**						
LR-1	L1_2_	10.33 (8.66)	17.61 (18.47)	19.71 (20.53)	17.63 (19.48)	34.73 (32.85)
LR-2	B2	26.72 (24.30)	11.22 (13.00)	3.04 (6.50)	10.11 (10.55)	48.91 (45.70)
LR-3	BCC	18.11	13.32	23.6	10.08	38.9
**NCR**						
NCR-1	L1_2_	9.29	19.01	19.58	19.46	32.65
NCR-2	FCC	2.73	24.00	23.94	23.97	25.34
NCR-3	B2	28.97	8.83	4.10	9.04	49.22
NCR-4	B2	8.69	5.43	61.87	7.60	16.39

**Figure 5:**
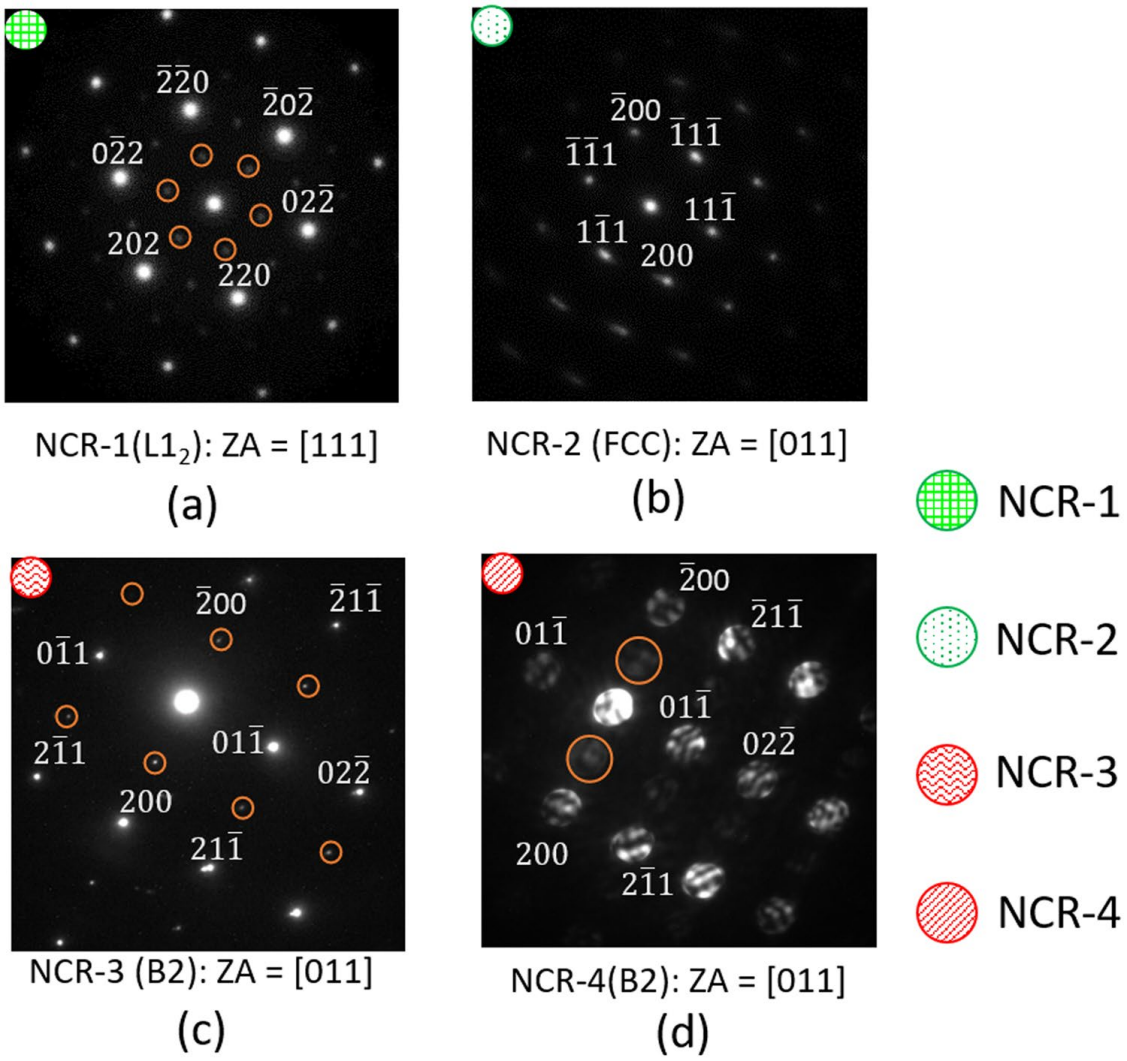
SADPs of (**a**) NCR-1 (L1_2_), (**b**) NCR-2 (disordered FCC), (**c**) NCR-3 (B2) and (**d**) NCR-4 (B2) are shown. The symbols present in the upper right corners in the SADPs correspond to the phases indicated by the identical symbols in Fig. 4(b).

The NCR shows remarkable contrast differences, indicating significant compositional variations (Fig. 4(b)). Based on the area mapping of the NCR in Fig. 4(b), the distribution of the constituent elements, namely Al (Fig. 4(c)), Co (Fig. 4(d)), Cr (Fig. 4(e)), Fe (Fig. 4(f)) and Ni (Fig. 4(g)), four different compositions appear to be present in the NCR. The grain marked NCR-1 has very similar composition to that of the L1_2_ phase in the LR (LR-1), as shown in Table 1. The SADP (Fig. 5(a)) shows ZA//[111] pattern of FCC with superlattice reflections, thus confirming the L1_2_ structure. The grain marked with the symbol NCR-2 in Fig. 4(b) is depleted in Al (Fig. 4(c)) as compared to the adjacent NCR-1, but shows nearly equal proportions of the other alloying elements (Table 1). The SADP (Fig. 5(b)) shows ZA//[011] FCC pattern without any superlattice reflections, thus confirming a disordered FCC structure of the NCR-2. The phase indicated by NCR-3 in Fig. 4(b) is rich in Ni and Al. The chemical composition is similar to that of the LR-2 (Table 1). The SADP (Fig. 5(c)) shows ZA//[011] BCC pattern with clearly distinguishable superlattice reflections, thus confirming the B2 structure. Finally, the phase marked by the symbol NCR-4 is remarkably rich in Cr (Fig. 4(e) and Table 1) showing a wide size range (~50 nm to 250 nm). The nano-beam electron diffraction (NBED) pattern (Fig. 5(d)) shows a ZA//[001] BCC pattern with distinguishable superlattice reflections, thus confirming the B2 structure of the Cr-rich phase.

## Discussion

The most striking aspect of the hybrid-rolled EHEA is the development of an extremely heterogeneous microstructure featured by the dual structural-compositional heterogeneities. The microstructural heterogeneities are featured by the existence of the LRs and NCRs, whereas the compositional heterogeneities are revealed by different phases with different crystal structures and compositions. Evidently, these extreme heterogeneities result from the unusual hybrid processing route. Therefore, the origin of the extremely heterogeneous microstructure and the outstanding mechanical properties of the hybrid-rolled EHEA remain the most important aspects of the present research.

**Figure 6:**
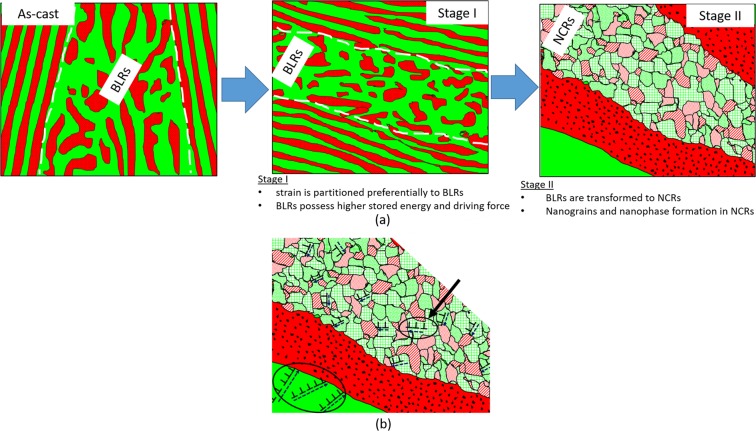
(**a**) Schematic illustration of the mechanism of evolution of structural-compositional dual heterogeneous microstructure in the EHEA during hybrid-rolling (Stage I: cryo-rolling and Stage II: warm-rolling). The NCRs are enclosed dotted lines (not to scale).; (**b**) shows the origin of long-range back stress in the heterogeneous EHEA. The colours of the various phase constituents are summarized in Table 1.

The origin of the NCRs can be related to the BLRs in the as-cast EHEA. BLRs are frequently reported in binary eutectics due to the instability in the lamellar growth^36^. In the Stage I deformation by cryo-rolling, the imposed strain is partitioned more to the BLRs than the LRs due to their more open/relaxed structure. The larger strain partitioning is corroborated by the deformation induced disordering and nano-crystallization of the L1_2_ in the BLRs. The higher stored energy of the BLRs provides the necessary driving force for the microstructural transformations during the subsequent warm-rolling (Stage II). The concomitant thermal energy and strain in Stage II deformation transforms the BLRs into NCRs. The NCRs show even further structural and chemical heterogeneities. The recrystallized regions are identified by lower KAM (volume fraction ~30%), while the deformed regions are evidenced by higher KAM and LAB network. The chemical heterogeneities are revealed by the presence of nanoscale phases with different compositions and crystal structures. This indicates that metastable microstructure of the as-cast EHEA breaks down during hybrid-rolling, accelerated and aided by the nanocrystalline structure of the NCR which enhances diffusion. Therefore, hybrid-rolling involves concomitant microstructural and phase transformations. Based on the mechanism as discussed, the origin of the novel heterogeneous microstructure is schematically illustrated in Fig. 6(a) for the ease of understanding.

The chemical compositions of NCR-1 (L1_2_) and NCR-3 (B2) show minor variations with the L1_2_ and B2 phases, respectively in the as-cast EHEA, indicating that they are retained from the as-cast state. While cryo-rolling (Stage I) results in the disordering of the L1_2_ phase, the ordered structure is recovered after warm-rolling (Stage II). This behaviour is rather similar to the L1_2_ Ni_3_Al, which is disordered by severe plastic deformation but recovered the ordering during annealing^37^. It appears that isothermal holding at 600 °C results in the rapid recovery of the ordered structure. The NCR-2 is depleted in Al but contains nearly equiatomic concentrations of Co, Cr, Fe and Ni. The disordered structure of the NCR-2 is in good agreement with the disordered FCC structure of the equiatomic quaternary CoCrFeNi. The B2 structured NCR-4 is remarkably rich in Cr and found mostly adjacent to the B2 structured NCR-2 phase. These phases could originate due to phase separation owing to compositional fluctuation, however, needs to be further investigated.

Development of ultrafine and nanostructured materials by severe plastic deformation processes have attracted considerable attention due to the possibility of extreme grain refinement and thereby achieving much superior mechanical strength. However, enhancing the usually poor ductility originating from the plastic strain instability and crack nucleation/growth instabilities without sacrificing tensile ductility remains a critical challenge. As reviewed recently by Ovid’ko *et al*.^38^, various strategies for improving the ductility have been proposed including grain boundaries deformation phenomena, introduction of nanotwins, formation of second phase particles and more recently through the formation of heterogeneous micro/nano-structure. While the extreme strength in the hybrid-processed EHEA is contributed by various strengthening mechanisms including nanostructuring and presence of hard B2 phase and nano-precipitates. The outstanding strength-ductility combination of the hybrid-rolled EHEA originates from the extreme heterogeneities. It is perceived that in heterogeneous materials, mechanical incompatibility between the different domains results in the development of strain gradients near the interfaces or domain boundaries, which must be accommodated by geometrically necessary dislocations (GNDs)^2,17^. The pile up of GNDs at the interfaces/domain boundaries leads to the development of long-range back stress which increases with the increasing strain, eventually leading to back-stress strengthening. The generation of such long-range back stress in the heterogeneous materials has been already experimentally verified^17,19,33^. The strain hardening due to the back stress is perceived to prevent the early onset of necking, rendering high strength and appreciable ductility.

The deformation in the present dual structural-compositional heterogeneous microstructure should be even more unique as schematically illustrated in Fig. 6(b). As already clarified, the B2 phase in the as-cast EHEA is much harder the L1_2_ phase^32,39^. Following the elastic deformation, the yielding of the softer L1_2_ lamellae is opposed by the hard B2 lamellae, resulting in the presence of the plastic strain gradients in the softer lamellae at the interface boundaries, which in turn are accommodated by the creation of GNDs and associated long range back stress, resulting in significant back stress strengthening (indicated by the enclosed circle in Fig. 6(b)). The NCRs with a nanocrystalline grains and phases sandwiched between the LRs should also give rise to plastic strain gradients and contribute to the generation of long-range back stress. Additionally, the NCRs are composed of several crystallographically and chemically different phases with expectedly varying hardness properties. Therefore, the same back stress is expected to originate in the NCR grains due to such complex phase mixtures (indicated by enclosed circle and marked by arrows in Fig. 6(b)), albeit at a possibly different scale and magnitude.

Unlike microstructural heterogeneities, which have been investigated so far to achieve strength-ductility synergy in different materials^2,3,17^ including HEAs^18,19,40–44^, the novel strategy developed in the present research leads to a different paradigm featured by dual structural-compositional heterogeneities. On the microstructural side, the heterogeneities are ensured by the presence of the LRs and NCRs, while on the compositional side it is featured by a number of different constituents. While the LRs consist of L1_2_ and B2 (containing disordered nano-precipitates), the NCRs contain different ordered and disordered phases with differences in structure, composition and internal strain or defect densities. Evidently, the dual structural-compositional heterogeneities offer a large number of interfaces and boundaries separating widely different hardness domains and phases. The massive back-stress strengthening in such heterogeneous microstructural arrangements results in super-strong EHEA coupled with appreciable ductility. The quantification of the back stress and its enhancement through further microstructural tuning in hybrid-processed dual structural-compositional heterogeneous EHEA remain topics of great interest and will be evaluated in the future work.

In summary, we have developed a unique dual structure-compositional heterogeneities EHEA using a novel hybrid-rolling route. The YS and UTS of the EHEAs are amongst the best for bulk structural materials. Together with the outstanding specific strength, the dual structure-compositional heterogeneous EHEA offers immense potential for advanced structural applications. It is envisaged that the novel hybrid processing route conceptualized and developed successfully in the present work could be judiciously exploited to tune the nanostructure and properties of a wide range of dual and multiphase HEAs with breakthrough properties.

## Materials and Methods

The AlCoCrFeNi_2.1_ EHEA was prepared by arc melting of high purity constituents (≥99.9%) in a Ti-gettered furnace. The melting process was repeated five times being suction-cast into a copper mould with dimensions of 90 mm (length) × 15 mm (width) × 3 mm (thickness). Samples with dimensions 20 mm (length) × 15 mm (width) × 3 mm (thickness) obtained from the suction cast ingot were used for the TMP processing. During hybrid-rolling, the samples were first multi-pass cryo-rolled (Stage I) to a thickness of ~1 mm (ε_cryo-rolling_ = 1.2). During cryo-rolling, the samples were immersed in a liquid N_2_ bath for 30 minutes before and immediately after each cryo-rolling pass. The Stage I deformation by cryo-rolling was followed by a multi-pass (a total number of ten passes were used) warm-rolling at 600 °C in Stage II (ε_warm-rolling_ = 1.2) to achieve the total strain (ε_total_ = ε_cryo-rolling_ + ε_warm-rolling_ = 2.4, equivalent to 90% thickness reduction with a final thickness of ~300 µm). During warm-rolling (Stage II), the specimens were held at 600 °C for 20 minutes prior to each warm-rolling pass and water quenched immediately after every pass. In order to highlight the effect of hybrid-rolling on mechanical properties of the EHEA, an as-cast EHEA specimen was monotonically cryo-rolled to the same true strain of ~2.4 corresponding to 90% reduction in thickness.

Electron backscatter diffraction (EBSD) system (Oxford Instruments, UK) attached to a FEG-SEM (Maker: Carl-Zeiss, Germany; Model: Supra 40) and transmission electron microscope (TEM) (Maker: JEOL, Japan, Model: JEM-2100) operated at 200 kV was used for microstructural characterization. The EBSD data was analysed by the TSL-OIM^TM^ software (EDAX Inc., USA). The chemical analysis was performed by the energy dispersive spectroscopy system ((EDAX Inc., USA) mounted on the TEM. The samples for EBSD and TEM (Φ = 3 mm disks) investigations were prepared by mechanical polishing followed by electro-polishing (temperature: −15 °C; applied voltage: 20 V; electrolyte: perchloric acid + ethanol 1:9 (by volume)). The tensile samples were extracted from the hybrid-rolled sheets and carefully polished before the tensile tests. Tensile properties were determined along the RD at ambient temperature using a universal testing machine (Shimadzu, Japan) with an initial strain rate of 8.3 × 10^−4^ s^−1^. The displacement of the gage section was accurately measured by a CCD video camera extensometer (SVS625MFCP), and the strain was calculated by the use of a standard digital image correlation (DIC) technique using VIC-2D software.

### Impact statement

A novel hybrid processing strategy achieves a unique structural-compositional dual heterogeneous microstructure in AlCoCrFeNi_2.1_ eutectic high entropy alloy resulting in unprecedented strength (>2000 MPa) and ductility (~8%) combination.

## Supplementary information

Dataset 1

## Data Availability

The dataset generated during and/or analysed during the current study are not publicly available as they form part of an ongoing study but are available from the corresponding author on reasonable request.
