# Supplementary material for: Nanostructuring with Structural-Compositional Dual Heterogeneities Enhances Strength-Ductility Synergy in Eutectic High Entropy Alloy
Source: Sci Rep. 2019 Aug 8;9:11505. doi: 10.1038/s41598-019-47983-y (PMC6687781; doi:10.1038/s41598-019-47983-y)
Supplement: Supplementary file 1 — Dataset 1 [file 41598_2019_47983_MOESM1_ESM.docx]

**Nanostructuring with Structural-Compositional Dual Heterogeneities Enhances Strength-Ductility Synergy in Eutectic High Entropy Alloy**

S.R. Reddy, S. Yoshida, T. Bhattacharjee, N. Sake, A. Lozinko, S. Guo, P.P. Bhattacharjee^*^, N. Tsuji

# Supplementary Table 1: Summary of the properties of the alloys plotted in Fig.1(b) and Fig.1(c) with their corresponding references.

| **S No.** | **Alloys** | **Processing Route** | **Phases present** | **Microstructure** | **YS (MPa)** | **UTS (MPa)** | **Elongation (%)** | **Ref.** |
| --- | --- | --- | --- | --- | --- | --- | --- | --- |
|  | AlCoCrCuFeNi | As-cast | BCC+2FCC | Coarse dendritic structure | 790 | 790 | 0 | 1 |
|  | AlCoCrCuFeNi | As-cast + 960°C/50h + multi-step forged at 950℃ | BCC+2FCC+σ | Fine equiaxed duplex structure | 1040 | 1170 | 1 | 1 |
|  | Al_0.5_CoCrCuFeNi | As-cast + 1000°C/6h/water quenching + cold rolling(80%) | 2FCC | Elongated grains with Cu-rich FCC network regions | 1292 | 1406 | 6 | 2,3 |
|  | Al_0.5_CoCrCuFeNi | As-cast + 1000°C/6h/water quenching + cold rolling(80%) + 900°C/10min | 2FCC | Nano crystalline grained FCC matrix with Cu-rich phase | 1021 | 1030 | 15 | 3 |
|  | Al_0.5_CoCrCuFeNi | As-cast + 1000°C/6h/water quenching + cold rolling(80%) + 900°C/30min | 2FCC | Ultrafine grain structure of FCC matrix with Cu-rich phase | 970 | 980 | 18 | 3 |
|  | Al_0.5_CoCrCuFeNi | As-cast + 1000°C/6h/water quenching + cold rolling(80%) + 900°C/60min | 2FCC | Ultrafine grain structure of FCC matrix with Cu-rich phase | 810 | 870 | 23 | 3 |
|  | Al_0.5_CoCrCuFeNi | As-cast + 1000°C/6h/water quenching + cold rolling(84%) | FCC + L12 | Two phase structure with α-FCC dendritic phase and β-FCC Cu-rich phase | 1284 | 1344 | 8 | 4 |
|  | Al_0.2_CoCrCu_0.2_FeNi_2_ | As-cast + 1200°C/24h + cold rolling(93%) + 700°C/20h/water quenching | FCC+L12 | FCC matrix with coherent L1_2_ (Ni,Cu)_3_Al nano precipitates | 719 | 1048 | 30 | 5 |
|  | Al_0.3_CoCrFeNi | As-cast + heat treated | FCC+B2 | Nano-sized B2 particles in FCC matrix | 1136 | 1207 | 8 | 6 |
|  | Al_0.5_CoCrFeNi | As-cast + 650°C/8 h/water quenching | FCC+BCC+B2 | Nano-sized B2 phase in inter dendritic region with precipitates in dendritic region | 834 | 1220 | 25 | 7 |
|  | AlCoCrFeNi_2.1_ | As-cast + cold rolling(90%) | FCC+B2 | Ultrafine disordered FCC + fragmented B phase | 1625 | 1800 | 6 | 8 |
|  | AlCoCrFeNi_2.1_ | As-cast + cold rolling(90%) + 800°C/1h | FCC+B2 | Ultra fine grained duplex structure of FCC and B2 phase | 1108 | 1200 | 12 | 9 |
|  | AlCoCrFeNi_2.1_ | As-cast + cold rolling(90%) + 1000°C/1h | FCC+B2 | Duplex structure of fine grained FCC + B2 | 844 | 1175 | 23 | 9 |
|  | Al_0.7_CoCrFe_2_Ni | As-cast | FCC+BCC+B2 | Coherent cuboidal B2 nano precipitates in Bcc matrix | 866 | 1223 | 8 | 10 |
|  | Al_0.096_CoCrNiTi_0.096_ | As-cast + 1200°C/2h/water quenching + cold rolling (66%) +  1160°C/3min/water quenching + 800°C/2h/water quenching | FCC+L12 | Nanoscale L1_2_ (Ni,Co,Cr)_3_(Ti,Al) | 750 | 1260 | 45 | 11 |
|  | AlCoCrFeNiTi | As-cast + 1200°C/4h + cold rolling(70%) + 650°C/4h/water quenching | FCC+L12+Ni2AlTi | L1_2_ Ni_3_(Ti,Al) coherent nano sized precipitates in FeCoNiCr FCC matrix | 1005 | 1273 | 17 | 12 |
|  | Al_0.2_CoFeNiSi_0.2_ | As-cast + cold rolling (60%) | FCC | Fe,Co rich Dendritic and Al,Ni,Si rich inter dendritic microstructure | 1149 | 1149 | 4 | 13 |
|  | Al_0.6_CoCrFeMnNi | As-cast | FCC+BCC+B2 | Duplex FCC+BCC/B2 structure | 832 | 1174 | 8 | 14 |
|  | Al_0.5_CrCuFeNi_2_ | As-cast + cold rolling(43%) + 900°C/24 h | BCC+FCC+ L12 | L1_2_ needle like structure + FCC solid solution with BCC phase | 704 | 1088 | 6 | 15 |
|  | Al_0.5_CrCuFeNi_2_ | As-cast + cold rolling (50%) | FCC | Polycrystalline FCC phase | 1055 | 1179 | 2 | 16 |
|  | AlCrFe_2_Ni_2_ | As-cast | FCC+BCC+B2 | Noodle like FCC phase with sphenoidal decomposed disordered BCC and ordered B2 phase | 796 | 1437 | 16 | 17 |
|  | Al_0.6_CrFe_2_Mn_1.2_Ni_0.8_ | As-cast | BCC+B2 | BCC matrix with homogeneously distributed cuboidal ordered B2 particles | 750 | 880 | 3 | 18 |
|  | CoCrFeMo_0.3_Ni | As-cast + cold rolling60% + 850°C/1h | FCC+σ+μ | FCC matrix with brittle intermetallic particles | 815 | 1186 | 19 | 19 |
|  | CoCr_1.3_FeMnNi_0.7_ | As-cast + 1000°C/24h + cold rolling80% + 675°C/1h | FCC+σ | FCC phase with minor σ-phase particles | 1153 | 1187 | 2 | 20 |
|  | CoCrFeMnNi | As-cast + 1000°C/24h + compressed 40% + 1000°C/1h + 77K cryo rolling 80% | FCC | Heavily twinned FCC phase with dense array of dislocations | 1500 | 1500 | 12 | 21 |
|  | CoCrFeMnNi | As-cast + 1000°C/24h + compressed 40% + 1000°C/1h + 293K cryo rolling 80% | FCC | Dislocation structure with cell boundaries | 1200 | 1200 | 14 | 21 |
|  | Co_0.19_Cr_0.08_Fe_1.54_Mn_1.04_Ni | As-cast + 900°C hot rolled (50%) + 1200°C/2h/water quenching + cold rolling64% | FCC | FCC phase with high dislocation densities | 760 | 760 | 17 | 22 |
|  | Co_1.5_CrFeNi_1.5_Ti_0.5_ | MA + SPS | 2FCC+BCC | Fine-twinned FCC grains with minor oxide inclusions | 1308 | 1384 | 4 | 23 |
|  | Co_1.5_CrFeNi_1.5_Ti_0.5_Mo_0.1_ | selective electron beam melting(SEBM) | FCC+Ni_3_Ti | Presence of FCC+Ni3Ti particles | 750 | 930 | 4 | 24 |
|  | Co_1.5_CrFeNi_1.5_Ti_0.5_Mo_0.1_ | Selective electron beam melting + 1120°C/3h/ air cooling | FCC+SC+ Ni_3_Ti | FCC matrix with distributed coarse Ni3Ti particles | 900 | 1320 | 18 | 24 |
|  | Co_1.5_CrFeNi_1.5_Ti_0.5_Mo_0.1_ | Selective electron beam melting + 1120°C/3h/water quenching | FCC+ SC+ Ni_3_Ti | FCC matrix with distributed fine Ni3Ti particles | 770 | 1120 | 36 | 24 |
|  | Co_1.75_Cr_0.75_FeMo_0.5_Ni | As-cast + 1200°C/48h + 1100°C Homogenized & cold rolling70% + 800°C/1h | FCC+μ | FCC solid solution with nano precipitates of Mo-rich µ phase | 1311 | 1410 | 12 | 25 |
|  | Co_1.75_Cr_0.75_FeMo_0.5_Ni | As-cast + 1200°C/48h + 1100°C Homogenized & cold rolling70% + 850°C/5min/water quenching | FCC+μ | FCC solid solution with nano precipitates of Mo-rich µ phase | 1212 | 1360 | 15 | 25 |
|  | Co_1.75_Cr_0.75_FeMo_0.5_Ni | As-cast + 1200°C/48h + 1100°C Homogenized & cold rolling70% + 900°C/5min/water quenching | FCC+μ | FCC solid solution with nano precipitates of Mo-rich µ phase | 1028 | 1249 | 18 | 25 |
|  | Co_1.75_Cr_0.75_FeMo_0.5_Ni | As-cast + 1200°C/48h + 1100°C Homogenized & cold rolling70% + 1000°C/5min/water quenching | FCC+μ | FCC solid solution with nano precipitates of Mo-rich µ phase | 879 | 1194 | 25 | 25 |
|  | Co_1.75_Cr_0.75_FeMo_0.5_Ni | As-cast + 1200°C/48h + 1100°C Homogenized & cold rolling70% + 1000°C/1h | FCC+μ | FCC solid solution with nano precipitates of Mo-rich µ phase | 799 | 1127 | 28 | 25 |
|  | Co_2.125_Cr_0.625_FeMo_0.25_Ni | As-cast + 1200°C/48h + 1100°C Homogenized & cold rolling70% + 700°C/1h | FCC | FCC solid solution with nano precipitates of Mo-rich µ phase | 800 | 1050 | 45 | 25 |
|  | HfNbTiZr | As-cast + 1300°C/6h | BCC | Single phase BCC structure | 879 | 969 | 15 | 26 |
|  | HfTaTiZr | As-cast | BCC | Single phase BCC with cast dendritic structure | 1356 | 1452 | 4 | 27 |
|  | HfTa_0.6_TiZr | As-cast | BCC | Single phase BCC structure | 750 | 1110 | 22 | 27 |
|  | HfTa_0.6_TiZr | As-cast | BCC+HCP | A Composite structure of BCC+HCP phase | 700 | 1119 | 30 | 27 |
|  | HfNbTaTiZr | As-cast | BCC | Coarse BCC phase with micro segregation in inter dendritic zones | 790 | 857 | 6 | 28 |
|  | HfNbTaTiZr | As-cast + hot isostatic pressing (HIP)@1200°C/207MPa/2h + 1200°C/24h + cold rolling 86% | BCC | Heavily deformed grain structure | 1202 | 1295 | 5 | 29 |
|  | HfNbTaTiZr | As-cast + HIP@1200°C/207MPa/2h + 1200°C/24h + cold rolling 86% + 800°C/2h | BCC | Partially recrystallized microstructure with heavily deformed grains and fine equiaxed grains | 1303 | 1334 | 2 | 29 |
|  | HfNbTaTiZr | As-cast + HIP@1200°C/207MPa/2h + 1200°C/24h + cold rolling86% + 1000°C/2h | BCC | Completely recrystallized microstructure with equiaxed grain morphology | 1145 | 1262 | 10 | 29 |
|  | Hf_0.5_Nb_0.5_Ta_0.5_Ti_1.5_Zr | As-cast | BCC | BCC single phase with segregation in dendritic strucutre | 903 | 990 | 19 | 30 |

# σ, µ, Ni_3_Ti are complex phases present in the respective materials

# Supplementary Table 1 References:

1. Kuznetsov, A. V. *et al.* Tensile properties of an AlCrCuNiFeCo high-entropy alloy in As-cast and wrought conditions. *Mater. Sci. Eng. A* **533**, 1017-118 (2012).
2. Tsai, C.W., Tsai, M.H., Yeh, J.W. & Yang, C.C. Effect of temperature on mechanical properties of Al_0.5_CoCrCuFeNi wrought alloy. *J. Alloy. Compd.* **490**, 160-165 (2010).
3. Tsai, C. W. *et al.* Microstructure and tensile properties of Al_0.5_CoCrCuFeNi alloys produced by simple rolling and annealing. *Mater. Sci. Technol.* **31**, 1178-1183 (2015).
4. Hemphill, M. A. *et al.* Fatigue behavior of Al_0.5_CoCrCuFeNi high entropy alloys. *Acta Mater.* **60**, 5723-5734 (2012).
5. Wang, Z. G. *et al.* Effect of coherent L1_2_ nanoprecipitates on the tensile behavior of a fcc-based high-entropy alloy. *Mater. Sci. Eng. A* **696**, 503-510 (2017).
6. Li, D. *et al.* High-entropy Al_0.3_CoCrFeNi alloy fibers with high tensile strength and ductility at ambient and cryogenic temperatures. *Acta Mater.* **123**, 285-294 (2017).
7. Niu, S. *et al.* Strengthening of nanoprecipitations in an annealed Al_0.5_CoCrFeNi high entropy alloy. *Mater. Sci. Eng. A* **671**, 82-86 (2016).
8. Wani, I. S. *et al.* Ultrafine-grained AlCoCrFeNi_2.1_ eutectic high-entropy alloy. *Mater. Res. Lett.* **4**, 174-179 (2016).
9. Wani, I. S. *et al.* Tailoring nanostructures and mechanical properties of AlCoCrFeNi_2.1_ eutectic high entropy alloy using thermo-mechanical processing. *Mater. Sci. Eng. A* **675**, 99-109 (2016).
10. Wang, Q. *et al.* A cuboidal B2 nanoprecipitation-enhanced body-centered-cubic alloy Al_0.7_CoCrFe_2_Ni with prominent tensile properties. *Scr. Mater.* **120**, 85-89 (2016).
11. Zhao, Y. L. *et al.* Heterogeneous precipitation behavior and stacking-fault-mediated deformation in a CoCrNi-based medium-entropy alloy. *Acta Mater.* **138**, 72-82 (2017).
12. He, J. Y. *et al.* A precipitation-hardened high-entropy alloy with outstanding tensile properties. *Acta Mater.* **102**, 187-196 (2016).
13. Zuo, T.T., Ren, S.B., Liaw, P. K. & Zhang, Y. Processing effects on the magnetic and mechanical properties of FeCoNiAl_0.2_Si_0.2_ high entropy alloy. *Int. J. Min. Met. Mater.* **20**, 549-555 (2013).
14. He, J. Y. *et al.* Effects of Al addition on structural evolution and tensile properties of the FeCoNiCrMn high-entropy alloy system. *Acta Mater.* **62**, 105-113 (2014).
15. Ng, C. *et al.* Phase stability and tensile properties of Co-free Al_0.5_CrCuFeNi_2_ high-entropy alloys. *J. Alloy. Compd.* **584**, 530-537 (2014).
16. Ma, S. G., Chen, Z. D. & Zhang, Y. Evolution of microstructures and properties of the Al_X_CrCuFeNi_2_ high-entropy alloys. *Mater. Sci. Forum.* **745-746**, 706-714 (2013).
17. Dong, Y. *et al.* A multi-component AlCrFe_2_Ni_2_ alloy with excellent mechanical properties. *Mater. Lett.* **169**, 62-64 (2016).
18. Shaysultanov, D. G. *et al.* Novel Fe_36_Mn_21_Cr_18_Ni_15_Al_10_ high entropy alloy with bcc/B2 dual-phase structure. *J. Alloy. Compd.* **705**, 756-763 (2017).
19. Liu, W. H. *et al.* Ductile CoCrFeNiMo_X_ high entropy alloys strengthened by hard intermetallic phases. *Acta Mater.* **116**, 332-342 (2016).
20. Zaddach, A. J., Scattergood, R. O. & Koch, C. C. Tensile properties of low-stacking fault energy high-entropy alloys. *Mater. Sci. Eng. A* **636**, 373-378 (2015).
21. Stepanov, N. *et al.* Effect of cryo-deformation on structure and properties of CoCrFeNiMn high-entropy alloy. *Intermetallics* **59**, 8-17 (2015).
22. Yao, M. J., Pradeep, K. G., Tasan, C. C. & Raabe, D. A novel, single phase, non-equiatomic FeMnNiCoCr high-entropy alloy with exceptional phase stability and tensile ductility. *Scr. Mater.* **72-73**, 5-8 (2014).
23. Moravcik, I. *et al.* Microstructure and mechanical properties of Ni_1.5_Co_1.5_CrFeTi_0.5_ high entropy alloy fabricated by mechanical alloying and spark plasma sintering. *Mater. Des.* **119**, 141-150 (2017).
24. Fujieda, T. *et al.* CoCrFeNiTi-based high-entropy alloy with superior tensile strength and corrosion resistance achieved by a combination of additive manufacturing using selective electron beam melting and solution treatment. *Mater. Lett.* **189**, 148-151 (2017).
25. Ming, K., Bi, X. & Wang, J. Precipitation strengthening of ductile Cr_15_Fe_20_Co_35_Ni_20_Mo_10_ alloys. *Scr. Mater.* **137**, 88-93 (2017).
26. Wu, Y. D. *et al.* A refractory Hf_25_Nb_25_Ti_25_Zr_25_ high-entropy alloy with excellent structural stability and tensile properties. *Mater. Lett.* **130**, 277-280 (2014).
27. Huang, H. *et al.* Phase-Transformation Ductilization of Brittle High-Entropy Alloys via Metastability Engineering. *Adv. Mater.* 1701678 *(*2017).
28. Dirras, G. *et al.* Elastic and plastic properties of As-cast equimolar TiHfZrTaNb high-entropy alloy. *Mater. Sci. Eng. A* **654**, 30-38 (2016).
29. Senkov, O. N. & Semiatin, S. L. Microstructure and properties of a refractory high-entropy alloy after cold working. *J. Alloy. Compd.* **649**, 1110-1123 (2015).
30. Sheikh, S. *et al.* Alloy design for intrinsically ductile refractory high-entropy alloys. *J. Appl. Phys.* **120**, 164902 (2016).

# Supplementary Table 2: Summary of the properties of the advanced alloys plotted in Fig.1 (d) with their corresponding references.

| **S No.** | **Material/Alloy** | **Processing Route** | **Phases present** | **Microstructure** | **YS (MPa)** | **Elongation (%)** | **Ref.** |
| --- | --- | --- | --- | --- | --- | --- | --- |
|  | SS 304 | surface mechanical attrition treatment (SMAT-H) | Austenite + α’ Martensite | Ultrafine grains to fine grains | 973 | 36 | 1 |
|  | TRIP 5 Mn | 1250 °C (2hr)+Forged+750 °C(30 min)+ 650 °C (144 hr) /air cooled | Austenite (γ) + Ferrite (α) | High fraction of globular γ/α grains and acicular γ/α grains | 845 | 24 | 2 |
|  | SS 304 | ECAP+625 °C (1 hr) | Austenite | equiaxed grains with sharp grain boundaries and annealing nanotwins, | 107 | 42 | 3 |
|  | Fe–Ni–Al–C  (HSS N05) | High pressure torsion (HPT) processed 0.5 rotations | Austenite+ Martenite | multimodal microstructures with γ + α’ crystal structures | 1303 | 21 | 4 |
|  | Fe–Ni–Al–C  (HSS N1) | HPT processed 1 rotations | Austenite+ Martenite | multimodal microstructures with γ + α’ crystal structures | 1685 | 23 | 4 |
|  | Fe–Ni–Al–C  (HSS N10) | HPT processed 10 rotations | Austenite+ Martenite | multimodal microstructures with γ + α’ crystal structures | 1810 | 20 | 4 |
|  | Fe-9Al-16Mn-C  (HSS 1) | cold rolling+900 °C (2 min)+water quenching | Austenite (γ)+ B2 | stringer bands + fine particles of size 200–1,000 nm +finer particles of size 50–300nm | 1351 | 20 | 5 |
|  | Fe-9Al-16Mn-C (HSS 2) | Cold Rolling+900 °C (8 min)+water quenching | Austenite (γ)+ B2 | stringer bands + fine particles of size 200–1,000 nm +finer particles of size 50–300nm | 1232 | 25 | 5 |
|  | Fe-9Al-16Mn-C (HSS 3) | Cold Rolling+900°C(15 min)+water quenching | Austenite (γ)+ B2 | stringer bands + fine particles of size 200–1,000 nm +finer particles of size 50–300nm | 1023 | 32 | 5 |
|  | Fe-11Al-20Mn-2C (TRIPLEX 1) | Cold rolling+ 1100 °C (15 min+water quenching | Austenite (γ)+ k-carbide | Austenite (γ) with fine kappa (k)-carbide precipitates | 1165 | 29 | 5 |
|  | TRIP 9 wt% Mn | 1100°C (Homogenized)+swaging(1000-1150°C)+ Hot rolled(1.3 true strain)+Cold rolled (1 true strain)+1050 (30 min)+ 450 °C(48 hr) | Martensite | coarse α’-martensite lamella | 975 | 16 | 6 |
|  | TRIP 12 wt% Mn | 1100°C (Homogenized)+swaging(1000-1150°C)+ Hot rolled(1.3 true strain)+Cold rolled (1 true strain)+1050 (30 min)+ 450 °C(48 hr) | Martensite+Asutenite | coarse α’-martensite + 15% retained austenite | 1317 | 11 | 6 |
|  | Fe–Mn–C(–Pd) TWIP steel | Swaged (CW 23%) | Austenite+ Pd rich precipitates | Austenite matrix with Pd rich fine precipitates | 1095 | 29 | 7 |

# Supplementary Table 2 References:

1. Chen, A. Y. et al. The influence of strain rate on the microstructure transition of 304 stainless steel. *Acta Mater.* **59**, 3697-3709 (2011).
2. Luo, H. et al. Experimental and numerical analysis on formation of stable austenite during the intercritical annealing of 5Mn steel. *Acta Mater.* **59**, 4002-4014 (2011).
3. Huang, C. X. et al. An Ideal Ultrafine-Grained Structure for High Strength and High Ductility. *Mater. Res. Lett.* **3**, 88-94 (2015).
4. Edalati, K., Furuta, T., Daio, T., Kuramoto, S. & Horita, Z. High Strength and High Uniform Ductility in a Severely Deformed Iron Alloy by Lattice Softening and Multimodal-structure Formation. *Mater. Res. Lett.* **3**, 197-202 (2015).
5. Kim, S.H., Kim, H. & Kim, N. J. Brittle intermetallic compound makes ultrastrong low-density steel with large ductility. *Nature* **518**, 77-79 (2015).
6. Raabe, D., Ponge, D., Dmitrieva, O. & Sander, B. Nanoprecipitate-hardened 1.5GPa steels with unexpected high ductility. *Scr. Mater.* **60**, 1141-1144 (2009).
7. Schinhammer, M. et al. Recrystallization behavior, microstructure evolution and mechanical properties of biodegradable Fe–Mn–C(–Pd) TWIP alloys. *Acta Mater.* **60**, 2746-2756 (2012).
